# Tetra­aquabis(6-carboxy-1*H*-benzimid­azole-5-carboxyl­ato-κ*N* ^3^)nickel(II) dimethyl­formamide disolvate dihydrate

**DOI:** 10.1107/S1600536809038069

**Published:** 2009-09-30

**Authors:** Hao Wang, Wen-Dong Song, Shi-Jie Li, Pei-Wen Qin, Shi-Wei Hu

**Affiliations:** a College of Food Science and Technology, Guang Dong Ocean University, Zhanjiang 524088, People’s Republic of China; b College of Science, Guang Dong Ocean University, Zhanjiang 524088, People’s Republic of China; c College of Agriculture, Guang Dong Ocean University, Zhanjiang 524088, People’s Republic of China

## Abstract

The title compound, [Ni(C_9_H_45_N_2_O_4_)_2_(H_2_O)_4_]·2C_3_H_7_NO·2H_2_O, has the Ni^II^ center coordinated by four water mol­ecules and two N atoms from two 1*H*-benzimidazole-5,6-dicarboxyl­ate ligands in an octa­hedral geometry. The mol­ecule inter­acts with the solvent water and dimethyl­formamide mol­ecules through N—H⋯O and O—H⋯O hydrogen bonds to form a three-dimensional supra­molecular network. The metal atom lies on a center of inversion.

## Related literature

For the crystal structures of 1*H*-benzimidazole-5,6-dicarboxyl­ate complexes, see: Gao *et al.* (2008 ▶); Lo *et al.* (2007 ▶); Song *et al.* (2009 ▶).
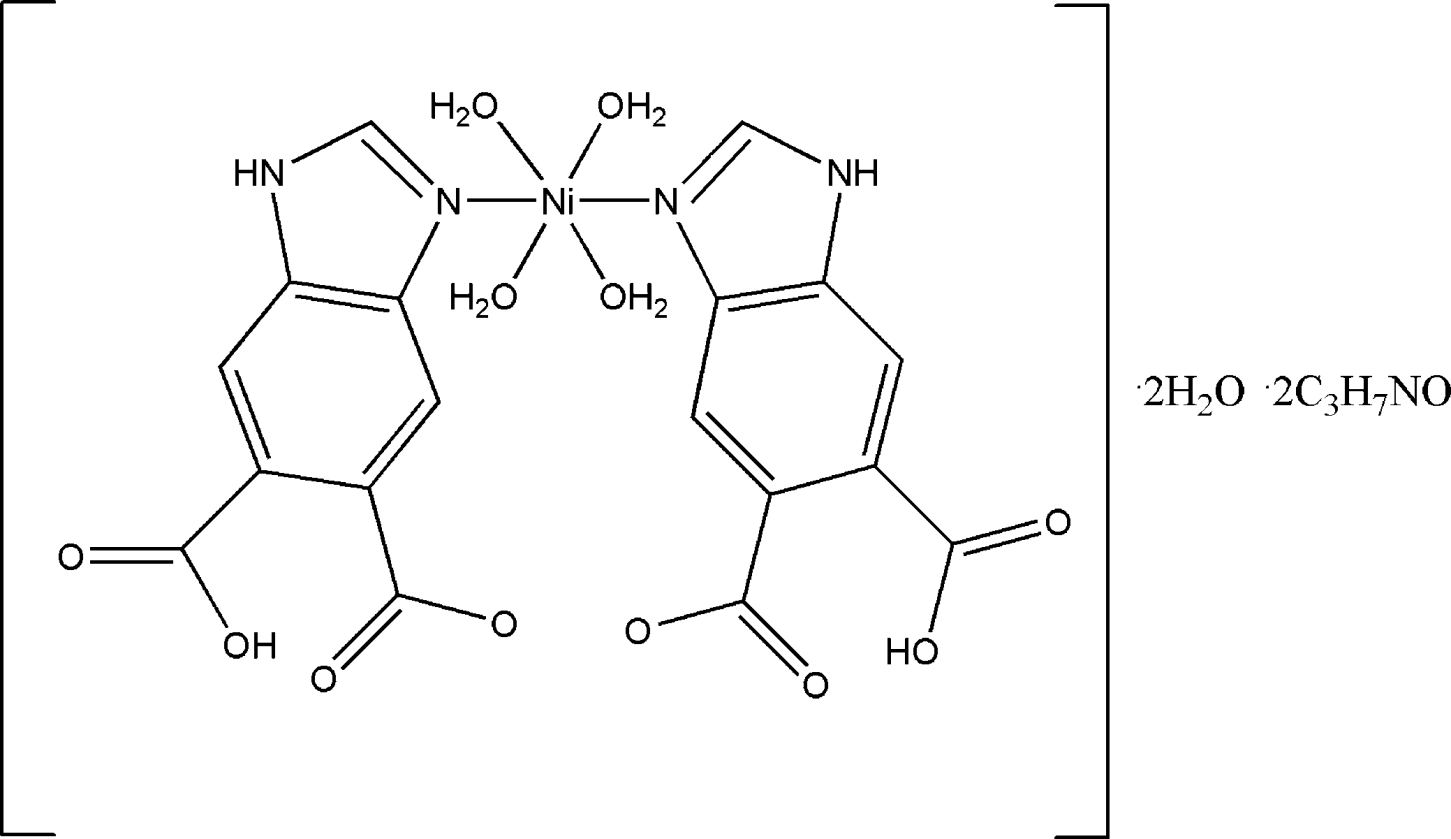


## Experimental

### 

#### Crystal data

[Ni(C_9_H_5_N_2_O_4_)_2_(H_2_O)_4_]·2C_3_H_7_NO·2H_2_O
*M* *_r_* = 723.30
Triclinic, 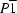

*a* = 8.5327 (17) Å
*b* = 9.1387 (18) Å
*c* = 11.624 (2) Å
α = 100.80 (3)°
β = 103.03 (3)°
γ = 114.04 (3)°
*V* = 765.7 (3) Å^3^
*Z* = 1
Mo *K*α radiation
μ = 0.72 mm^−1^
*T* = 293 K
0.27 × 0.18 × 0.17 mm

#### Data collection

Rigaku/MSC Mercury CCD diffractometer
Absorption correction: multi-scan (*REQAB*; Jacobson, 1998 ▶) *T*_min_ = 0.830, *T*_max_ = 0.888
6116 measured reflections
2737 independent reflections
2613 reflections with *I* > 2σ(*I*)
*R*_int_ = 0.020

#### Refinement

*R*[*F* ^2^ > 2σ(*F* ^2^)] = 0.037
*wR*(*F* ^2^) = 0.137
*S* = 1.20
2737 reflections
217 parameters
9 restraints
H-atom parameters constrained
Δρ_max_ = 0.61 e Å^−3^
Δρ_min_ = −0.32 e Å^−3^

### 

Data collection: *RAPID-AUTO* (Rigaku, 1998 ▶); cell refinement: *RAPID-AUTO*; data reduction: *CrystalStructure* (Rigaku/MSC, 2002 ▶); program(s) used to solve structure: *SHELXS97* (Sheldrick, 2008 ▶); program(s) used to refine structure: *SHELXL97* (Sheldrick, 2008 ▶); molecular graphics: *ORTEPII* (Johnson, 1976 ▶); software used to prepare material for publication: *SHELXL9*.

## Supplementary Material

Crystal structure: contains datablocks I, global. DOI: 10.1107/S1600536809038069/ng2646sup1.cif

Structure factors: contains datablocks I. DOI: 10.1107/S1600536809038069/ng2646Isup2.hkl

Additional supplementary materials:  crystallographic information; 3D view; checkCIF report

## Figures and Tables

**Table 1:** Hydrogen-bond geometry (Å, °)

*D*—H⋯*A*	*D*—H	H⋯*A*	*D*⋯*A*	*D*—H⋯*A*
O1*W*—H2*W*⋯O1^i^	0.84	1.86	2.693 (3)	173
O1*W*—H1*W*⋯O3^ii^	0.84	2.00	2.801 (3)	160
O4—H4*A*⋯O5^iii^	0.82	1.78	2.585 (3)	167
O2*W*—H4*W*⋯O2^iv^	0.84	1.79	2.624 (3)	176
O2*W*—H3*W*⋯O1*W*^v^	0.84	1.92	2.741 (2)	166
O3*W*—H5*W*⋯O1*W*^v^	0.84	2.06	2.810 (3)	148
O3*W*—H6*W*⋯O1^vi^	0.84	1.81	2.634 (3)	169
N2—H2⋯O5^vii^	0.86	1.98	2.779 (3)	155

Symmetry codes: (i) 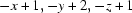; (ii) 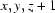; (iii) 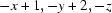; (iv) 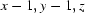; (v) 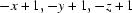; (vi) 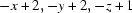; (vii) 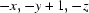.

**Fig. 1.**
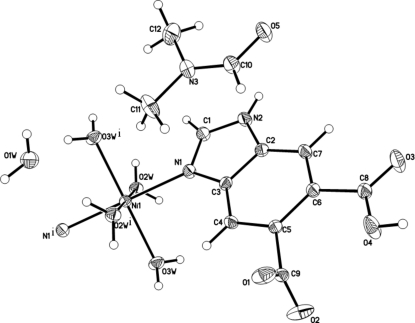
The structure of the title compound, showing the atomic numbering scheme with 30% probability displacement ellipsoids. [Symmetry codes: (i) 1 - x, 1 - y, 1 - z.]

**Fig. 2.**
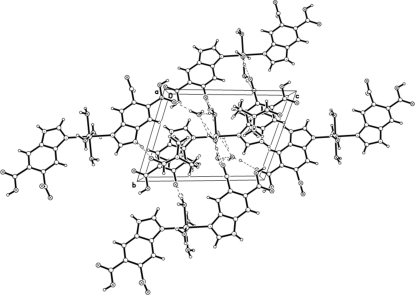
A view of the three-dimensional network constructed by O—H···O and N—H···O hydrogen bonding interactions.

## supplementary crystallographic information

### Comment

From the structuralpoint of view, 1*H*-benzimidazole-5,6-dicarboxylic acid possesses two nitrogen atoms of imidazole ring and four oxygen atoms of carboxylate groups, and might be used as versatile linker in constructing coordination polymers with abundant hydrogen bonds. And several coordination polymers fomed by this ligand have been reported recently:Pentaaqua(1*H*-benzimidazole-5,6-dicarboxylato-kN^3^)copper(II) pentahydrate(Gao *et al.*,2008), Bis(1*H*-benzimidazole-5,6-dicarboxylato)bis[tetraaquadicobalt(II)] pentahydrate(Lo *et al.*,2007), Pentaaqua(1*H*-benzimidazole-5,6-dicarboxylato-kN^3^) cobalt(II)pentahydrate(Song *et al.*,2009).In the present paper, we synthesized a novel coordination complex [Ni(C_9_H_4_N_2_O_4_)_2_(H_2_O)_4_].2H_2_O.2C_3_H_7_NO.

As shown in Figure 1, the Ni^II^ atom exhibits an octahedral coordination sphere, defined by two N atoms from two different 1*H*-benzimidazole-5,6-dicarboxylate ligands, and four water molecules. The equatorial plane is defined by O2w, O3w, O2w^i^ and O3w^i^ atoms, while N1 and N1^i^ occupy the axial position (symmetry codes: i = 1 - *x*, 1 - *y*, 1 - *z*). Inter/intramolecular O—H···O and N—H···O hydrogen bonds between the carboxylate O atoms of 1*H*-benzimidazole-5,6-dicarboxylate and the coordinated water molecule lead to the structure more stable(Fig 2).The hydrogen bonds are in the normal range(Table 1).

### Experimental

A C_3_H_7_NO solution (20 mL)containing Ni(NO_3_)_2_(0.1 mmol)and 1*H*-benzimidazole-5,6-dicarboxylic acid(0.2 mmol) was stirred for a few minutes in air,and left to stand at room temperature for about four weeks, then the green crystals were obtained.

### Refinement

Carbon and nitrogen bound H atoms were placed at calculated positions and were treated as riding on the parent C or N atoms with C—H = 0.93 Å, N—H = 0.86 Å, and with *U*_iso_(H) = 1.2 *U*_eq_(C, N). The water H-atoms were located in a difference map, and were refined with a distance restraint of O—H = 0.84 Å; their *U*_iso_ values were refined.

### Crystal data

**Table:**

[Ni(C_9_H_5_N_2_O_4_)_2_(H_2_O)_4_]·2C_3_H_7_NO·2H_2_O	*Z* = 1
*M**_r_* = 723.30	*F*(000) = 378
Triclinic, *P*1	*D*_x_ = 1.569 Mg m^−^^3^
Hall symbol: -P 1	Mo *K*α radiation, λ = 0.71073 Å
*a* = 8.5327 (17) Å	Cell parameters from 3600 reflections
*b* = 9.1387 (18) Å	θ = 1.4–28°
*c* = 11.624 (2) Å	µ = 0.72 mm^−^^1^
α = 100.80 (3)°	*T* = 293 K
β = 103.03 (3)°	Block, green
γ = 114.04 (3)°	0.27 × 0.18 × 0.17 mm
*V* = 765.7 (3) Å^3^	

### Data collection

**Table:**

Rigaku/MSC Mercury CCD diffractometer	2737 independent reflections
Radiation source: fine-focus sealed tube	2613 reflections with *I* > 2σ(*I*)
graphite	*R*_int_ = 0.020
ω scans	θ_max_ = 25.2°, θ_min_ = 3.3°
Absorption correction: multi-scan (REQAB; Jacobson, 1998)	*h* = −10→10
*T*_min_ = 0.830, *T*_max_ = 0.888	*k* = −10→9
6116 measured reflections	*l* = −13→13

### Refinement

**Table:**

Refinement on *F*^2^	Primary atom site location: structure-invariant direct methods
Least-squares matrix: full	Secondary atom site location: difference Fourier map
*R*[*F*^2^ > 2σ(*F*^2^)] = 0.037	Hydrogen site location: inferred from neighbouring sites
*wR*(*F*^2^) = 0.137	H-atom parameters constrained
*S* = 1.20	*w* = 1/[σ^2^(*F*_o_^2^) + (0.1051*P*)^2^ + 0.01*P*] where *P* = (*F*_o_^2^ + 2*F*_c_^2^)/3
2737 reflections	(Δ/σ)_max_ < 0.001
217 parameters	Δρ_max_ = 0.61 e Å^−^^3^
9 restraints	Δρ_min_ = −0.32 e Å^−^^3^

### Special details

**Table:**

Geometry. All e.s.d.'s (except the e.s.d. in the dihedral angle between two l.s. planes) are estimated using the full covariance matrix. The cell e.s.d.'s are taken into account individually in the estimation of e.s.d.'s in distances, angles and torsion angles; correlations between e.s.d.'s in cell parameters are only used when they are defined by crystal symmetry. An approximate (isotropic) treatment of cell e.s.d.'s is used for estimating e.s.d.'s involving l.s. planes.
Refinement. Refinement of *F*^2^ against ALL reflections. The weighted *R*-factor *wR* and goodness of fit *S* are based on *F*^2^, conventional *R*-factors *R* are based on *F*, with *F* set to zero for negative *F*^2^. The threshold expression of *F*^2^ > σ(*F*^2^) is used only for calculating *R*-factors(gt) *etc*. and is not relevant to the choice of reflections for refinement. *R*-factors based on *F*^2^ are statistically about twice as large as those based on *F*, and *R*- factors based on ALL data will be even larger.

### Fractional atomic coordinates and isotropic or equivalent isotropic displacement parameters (Å^2^)

**Table:**

	*x*	*y*	*z*	*U*_iso_*/*U*_eq_	
C1	0.2895 (3)	0.4030 (3)	0.2261 (2)	0.0301 (5)	
H1	0.2030	0.3062	0.2339	0.036*	
N1	0.4387 (2)	0.5153 (2)	0.31852 (17)	0.0272 (4)	
Ni1	0.5000	0.5000	0.5000	0.02200 (19)	
C2	0.4271 (3)	0.5938 (3)	0.1432 (2)	0.0272 (5)	
N2	0.2739 (2)	0.4415 (2)	0.11945 (17)	0.0324 (4)	
H2	0.1861	0.3832	0.0499	0.039*	
C3	0.5296 (3)	0.6391 (3)	0.2686 (2)	0.0252 (4)	
O3W	0.75996 (16)	0.54366 (16)	0.50333 (12)	0.0345 (4)	
C4	0.6923 (3)	0.7892 (3)	0.3224 (2)	0.0271 (5)	
H4	0.7612	0.8210	0.4056	0.033*	
C5	0.7498 (3)	0.8909 (3)	0.2494 (2)	0.0248 (4)	
O1	0.9319 (2)	1.1775 (2)	0.37262 (18)	0.0456 (5)	
C6	0.6429 (3)	0.8423 (3)	0.1223 (2)	0.0280 (5)	
O2	1.0690 (2)	1.0376 (2)	0.3063 (2)	0.0522 (5)	
C7	0.4811 (3)	0.6925 (3)	0.0690 (2)	0.0307 (5)	
H7	0.4113	0.6596	−0.0141	0.037*	
C8	0.6944 (3)	0.9451 (3)	0.0384 (2)	0.0327 (5)	
C9	0.9321 (3)	1.0500 (3)	0.31293 (19)	0.0261 (5)	
O2W	0.41483 (16)	0.24688 (15)	0.42925 (12)	0.0295 (4)	
H6W	0.8553	0.6369	0.5347	0.044*	
H5W	0.7568	0.4874	0.4361	0.044*	
H3W	0.4755	0.2330	0.3845	0.044*	
H4W	0.3028	0.1830	0.3922	0.044*	
O3	0.6082 (3)	0.8962 (3)	−0.07127 (17)	0.0591 (6)	
O4	0.8343 (3)	1.0927 (3)	0.09199 (18)	0.0633 (7)	
H4A	0.8481	1.1446	0.0415	0.095*	
O1W	0.3424 (2)	0.7464 (3)	0.69171 (17)	0.0487 (5)	
H1W	0.3996	0.7866	0.7687	0.073*	
H2W	0.2637	0.7784	0.6716	0.073*	
O5	0.0718 (2)	0.7407 (2)	0.05904 (16)	0.0462 (5)	
C12	−0.0319 (4)	0.5721 (4)	0.2255 (3)	0.0570 (8)	
H12A	−0.1264	0.6015	0.2316	0.086*	
H12B	−0.0161	0.5128	0.2835	0.086*	
H12C	−0.0653	0.5013	0.1426	0.086*	
N3	0.1376 (3)	0.7248 (3)	0.25427 (18)	0.0352 (5)	
C11	0.2581 (4)	0.8066 (4)	0.3847 (2)	0.0522 (7)	
H11A	0.3625	0.9074	0.3914	0.078*	
H11B	0.2971	0.7308	0.4129	0.078*	
H11C	0.1933	0.8351	0.4351	0.078*	
C10	0.1722 (3)	0.7970 (3)	0.1704 (2)	0.0356 (5)	
H10	0.2794	0.8978	0.1949	0.043*	

### Atomic displacement parameters (Å^2^)

**Table:**

	*U*^11^	*U*^22^	*U*^33^	*U*^12^	*U*^13^	*U*^23^
C1	0.0264 (11)	0.0250 (11)	0.0280 (11)	0.0029 (9)	0.0068 (9)	0.0092 (9)
N1	0.0272 (9)	0.0227 (9)	0.0259 (9)	0.0057 (7)	0.0082 (7)	0.0098 (7)
Ni1	0.0196 (3)	0.0191 (3)	0.0217 (3)	0.00424 (18)	0.00625 (17)	0.00652 (17)
C2	0.0216 (10)	0.0230 (10)	0.0262 (11)	0.0024 (8)	0.0062 (8)	0.0055 (9)
N2	0.0256 (9)	0.0274 (10)	0.0254 (9)	−0.0008 (8)	0.0021 (7)	0.0071 (8)
C3	0.0242 (10)	0.0248 (10)	0.0264 (11)	0.0093 (8)	0.0112 (8)	0.0099 (9)
O3W	0.0231 (8)	0.0295 (8)	0.0380 (9)	0.0049 (6)	0.0105 (6)	0.0003 (7)
C4	0.0231 (10)	0.0266 (11)	0.0223 (10)	0.0059 (8)	0.0037 (8)	0.0060 (8)
C5	0.0223 (10)	0.0237 (11)	0.0245 (10)	0.0075 (8)	0.0074 (8)	0.0078 (8)
O1	0.0282 (9)	0.0305 (9)	0.0559 (11)	0.0043 (7)	0.0102 (7)	−0.0071 (8)
C6	0.0252 (10)	0.0278 (11)	0.0258 (11)	0.0073 (9)	0.0082 (8)	0.0098 (9)
O2	0.0233 (9)	0.0324 (9)	0.0833 (15)	0.0073 (7)	0.0126 (8)	0.0002 (9)
C7	0.0283 (11)	0.0312 (11)	0.0217 (10)	0.0069 (9)	0.0037 (8)	0.0072 (9)
C8	0.0295 (11)	0.0328 (12)	0.0266 (12)	0.0063 (9)	0.0078 (9)	0.0113 (10)
C9	0.0220 (10)	0.0249 (11)	0.0249 (11)	0.0059 (9)	0.0050 (8)	0.0095 (9)
O2W	0.0250 (7)	0.0233 (7)	0.0316 (8)	0.0058 (6)	0.0076 (6)	0.0054 (6)
O3	0.0561 (12)	0.0527 (12)	0.0269 (9)	−0.0082 (9)	0.0002 (8)	0.0191 (9)
O4	0.0599 (12)	0.0464 (11)	0.0327 (10)	−0.0161 (9)	−0.0030 (9)	0.0224 (9)
O1W	0.0396 (10)	0.0590 (12)	0.0390 (10)	0.0258 (9)	0.0087 (8)	−0.0036 (9)
O5	0.0409 (10)	0.0468 (10)	0.0272 (9)	0.0019 (8)	0.0025 (7)	0.0156 (8)
C12	0.0560 (17)	0.0591 (18)	0.0607 (19)	0.0195 (14)	0.0312 (15)	0.0326 (15)
N3	0.0421 (11)	0.0367 (11)	0.0260 (10)	0.0184 (9)	0.0101 (9)	0.0103 (9)
C11	0.078 (2)	0.0531 (17)	0.0285 (13)	0.0379 (16)	0.0077 (13)	0.0140 (12)
C10	0.0363 (12)	0.0301 (12)	0.0302 (12)	0.0083 (10)	0.0065 (10)	0.0104 (10)

### Geometric parameters (Å, °)

**Table:**

C1—N1	1.317 (3)	C6—C7	1.386 (3)
C1—N2	1.344 (3)	C6—C8	1.492 (3)
C1—H1	0.9300	O2—C9	1.236 (3)
N1—C3	1.397 (3)	C7—H7	0.9300
N1—Ni1	2.1014 (18)	C8—O3	1.209 (3)
Ni1—O2W	2.0501 (14)	C8—O4	1.293 (3)
Ni1—O2W^i^	2.0501 (14)	O2W—H3W	0.8401
Ni1—O3W^i^	2.0773 (13)	O2W—H4W	0.8400
Ni1—O3W	2.0773 (13)	O4—H4A	0.8200
Ni1—N1^i^	2.1014 (18)	O1W—H1W	0.8408
C2—C7	1.379 (3)	O1W—H2W	0.8405
C2—N2	1.390 (3)	O5—C10	1.252 (3)
C2—C3	1.401 (3)	C12—N3	1.454 (4)
N2—H2	0.8600	C12—H12A	0.9600
C3—C4	1.391 (3)	C12—H12B	0.9600
O3W—H6W	0.8402	C12—H12C	0.9600
O3W—H5W	0.8394	N3—C10	1.298 (3)
C4—C5	1.391 (3)	N3—C11	1.470 (3)
C4—H4	0.9300	C11—H11A	0.9600
C5—C6	1.424 (3)	C11—H11B	0.9600
C5—C9	1.522 (3)	C11—H11C	0.9600
O1—C9	1.240 (3)	C10—H10	0.9300
			
N1—C1—N2	113.71 (18)	C4—C5—C9	115.96 (18)
N1—C1—H1	123.1	C6—C5—C9	123.63 (19)
N2—C1—H1	123.1	C7—C6—C5	120.7 (2)
C1—N1—C3	104.99 (18)	C7—C6—C8	115.74 (19)
C1—N1—Ni1	123.63 (15)	C5—C6—C8	123.59 (19)
C3—N1—Ni1	131.30 (15)	C2—C7—C6	117.85 (19)
O2W—Ni1—O2W^i^	180.0	C2—C7—H7	121.1
O2W—Ni1—O3W^i^	91.85 (6)	C6—C7—H7	121.1
O2W^i^—Ni1—O3W^i^	88.15 (6)	O3—C8—O4	122.2 (2)
O2W—Ni1—O3W	88.15 (6)	O3—C8—C6	122.5 (2)
O2W^i^—Ni1—O3W	91.85 (6)	O4—C8—C6	115.3 (2)
O3W^i^—Ni1—O3W	180.0	O2—C9—O1	125.6 (2)
O2W—Ni1—N1^i^	90.06 (7)	O2—C9—C5	116.7 (2)
O2W^i^—Ni1—N1^i^	89.94 (7)	O1—C9—C5	117.58 (18)
O3W^i^—Ni1—N1^i^	90.14 (7)	Ni1—O2W—H3W	109.0
O3W—Ni1—N1^i^	89.86 (7)	Ni1—O2W—H4W	117.7
O2W—Ni1—N1	89.94 (7)	H3W—O2W—H4W	110.9
O2W^i^—Ni1—N1	90.06 (7)	C8—O4—H4A	109.5
O3W^i^—Ni1—N1	89.86 (7)	H1W—O1W—H2W	111.4
O3W—Ni1—N1	90.14 (7)	N3—C12—H12A	109.5
N1^i^—Ni1—N1	180.0	N3—C12—H12B	109.5
C7—C2—N2	131.9 (2)	H12A—C12—H12B	109.5
C7—C2—C3	122.56 (19)	N3—C12—H12C	109.5
N2—C2—C3	105.48 (19)	H12A—C12—H12C	109.5
C1—N2—C2	106.79 (18)	H12B—C12—H12C	109.5
C1—N2—H2	126.6	C10—N3—C12	121.1 (2)
C2—N2—H2	126.6	C10—N3—C11	120.7 (2)
C4—C3—N1	131.2 (2)	C12—N3—C11	117.8 (2)
C4—C3—C2	119.76 (19)	N3—C11—H11A	109.5
N1—C3—C2	109.02 (19)	N3—C11—H11B	109.5
Ni1—O3W—H6W	126.3	H11A—C11—H11B	109.5
Ni1—O3W—H5W	111.1	N3—C11—H11C	109.5
H6W—O3W—H5W	111.6	H11A—C11—H11C	109.5
C5—C4—C3	118.77 (19)	H11B—C11—H11C	109.5
C5—C4—H4	120.6	O5—C10—N3	124.8 (2)
C3—C4—H4	120.6	O5—C10—H10	117.6
C4—C5—C6	120.39 (19)	N3—C10—H10	117.6

Symmetry codes: (i) −*x*+1, −*y*+1, −*z*+1.

### Hydrogen-bond geometry (Å, °)

**Table:**

*D*—H···*A*	*D*—H	H···*A*	*D*···*A*	*D*—H···*A*
O1W—H2W···O1^ii^	0.84	1.86	2.693 (3)	173
O1W—H1W···O3^iii^	0.84	2.00	2.801 (3)	160
O4—H4A···O5^iv^	0.82	1.78	2.585 (3)	167
O2W—H4W···O2^v^	0.84	1.79	2.624 (3)	176
O2W—H3W···O1W^i^	0.84	1.92	2.741 (2)	166
O3W—H5W···O1W^i^	0.84	2.06	2.810 (3)	148
O3W—H6W···O1^vi^	0.84	1.81	2.634 (3)	169
N2—H2···O5^vii^	0.86	1.98	2.779 (3)	155

Symmetry codes: (ii) −*x*+1, −*y*+2, −*z*+1; (iii) *x*, *y*, *z*+1; (iv) −*x*+1, −*y*+2, −*z*; (v) *x*−1, *y*−1, *z*; (i) −*x*+1, −*y*+1, −*z*+1; (vi) −*x*+2, −*y*+2, −*z*+1; (vii) −*x*, −*y*+1, −*z*.
